# Focal Adhesion Kinase: Insight into Molecular Roles and Functions in Hepatocellular Carcinoma

**DOI:** 10.3390/ijms18010099

**Published:** 2017-01-05

**Authors:** Nadia Panera, Annalisa Crudele, Ilaria Romito, Daniela Gnani, Anna Alisi

**Affiliations:** Liver Research Unit, Bambino Gesù Children’s Hospital, IRCCS, Via S. Paolo, 15, 00146 Rome, Italy; nadia.panera@opbg.net (N.P.); annalisa.crudele@opbg.net (A.C.); ilaria.romito@opbg.net (I.R.); daniela.gnani@yahoo.it (D.G.)

**Keywords:** focal adhesion kinase, hepatocellular carcinoma, cancer stem cells, proliferation, metastasis

## Abstract

Hepatocellular carcinoma (HCC) is the third leading cause of cancer-related death worldwide. Due to the high incidence of post-operative recurrence after current treatments, the identification of new and more effective drugs is required. In previous years, new targetable genes/pathways involved in HCC pathogenesis have been discovered through the help of high-throughput sequencing technologies. Mutations in *TP53* and β-catenin genes are the most frequent aberrations in HCC. However, approaches able to reverse the effect of these mutations might be unpredictable. In fact, if the reactivation of proteins, such as p53 in tumours, holds great promise as anticancer therapy, there are studies arguing that chronic activation of these types of molecules may be deleterious. Thus, recently the efforts on potential targets have focused on actionable mutations, such as those occurring in the gene encoding for focal adhesion kinase (FAK). This tyrosine kinase, localized to cellular focal contacts, is over-expressed in a variety of human tumours, including HCC. Moreover, several lines of evidence demonstrated that FAK depletion or inhibition impair in vitro and in vivo HCC growth and metastasis. Here, we provide an overview of FAK expression and activity in the context of tumour biology, discussing the current evidence of its connection with HCC development and progression.

## 1. Introduction

Hepatocellular carcinoma (HCC) is one of the most common human malignancies, which accounts for 70%–85% of the primary tumours of the liver and is the third leading cause of cancer-related death worldwide [1,2]. Over the past 20 years, the research in the field of HCC has progressed very quickly, improving diagnosis and treatment of this type of tumour [2]. In fact, current effective therapies for HCC, including liver resection, local ablation, and transplantation, may result in five-year survival rates up to 75% [3,4]. However, liver transplantation in HCC is limited by the cost and availability of donor organs, while, with the surgical approach, there is still a high incidence of post-operative recurrence and a high propensity to cause intra-hepatic metastasis [5,6]. Therefore, it is crucial to find new and more effective treatments to counteract HCC. An in-depth knowledge of mechanisms, which drive HCC development and progression, could make a substantial contribution here.

It is well known that liver fibrosis is strongly associated with HCC, with 90% of HCC cases arising in cirrhotic livers, while the remaining cases occur in a non-cirrhotic background [7,8,9]. The main risk factors for HCC development are viral hepatitis B and C, alcohol abuse, and aflatoxin B1 exposure [1]. However, it is now clear that other factors, such as non-alcoholic fatty liver disease, diabetes, obesity, diet and hemochromatosis, may also predispose liver to HCC development, especially in industrialized countries [1,8]. The hepatic damage, induced by these factors, may trigger extensive cellular apoptosis and inflammation [7,8]. In these conditions, the physiological regenerative capacity of the hepatocytes can be compromised, and uncontrolled proliferation of a cancer stem cell component may take place, enhancing post-resection tumour recurrence due to the effects of acute injury [10].

HCC is characterized by the accumulation of genetic mutations and epigenetic changes occurring during onset, promotion, and progression of the tumour [11,12,13]. In fact, previous studies have revealed that mutations in telomerase reverse transcriptase (*TERT*), *TP53*, and β-catenin genes (*CTNNB1*) frequently occur in HCC [13]. Moreover, in the last years, new genes/pathways implicated in liver cancer have been discovered through the help of high-throughput sequencing technologies [14,15]. Interestingly, among the new genes, emerged the protein tyrosine kinase 2 (*PTK2*) gene encoding for the focal adhesion kinase (FAK). Indeed, it has been reported that approximately 26.1% of HCCs harbour *PTK2* gene amplification [15]. FAK is a tyrosine kinase, mainly localized to cellular focal contacts, over-expressed in a variety of human tumours, including HCC, suggesting a potential role of this protein in tumour formation and malignant progression [16,17,18]. This could be of great relevance for anticancer therapy against HCC where FAK has been described as a clinically actionable mutation [15].

In this review article, we provide an overview of FAK expression and activity in the context of tumour biology, and we discuss the current evidence of the role of this protein in HCC development and progression, suggesting its potential use as a therapeutic target.

## 2. Structure and Functions of FAK

### 2.1. FAK Functional Domains

FAK is a highly conserved 125 kDa non-receptor tyrosine kinase that plays a critical role in adhesion-dependent cell motility, survival, and proliferation in response to integrin and growth factor receptor signalling [19]. For a long time, FAK was considered as a simple sensor of environmental rigidity [20,21]. Nowadays, different researchers have found that FAK may exert specific functions depending on different subcellular environments [19]. In fact, FAK protein is involved in an intricate network of intramolecular interactions existing between the microenvironment, the adhesion receptor complexes and the nucleus [22].

**Figure 1:**
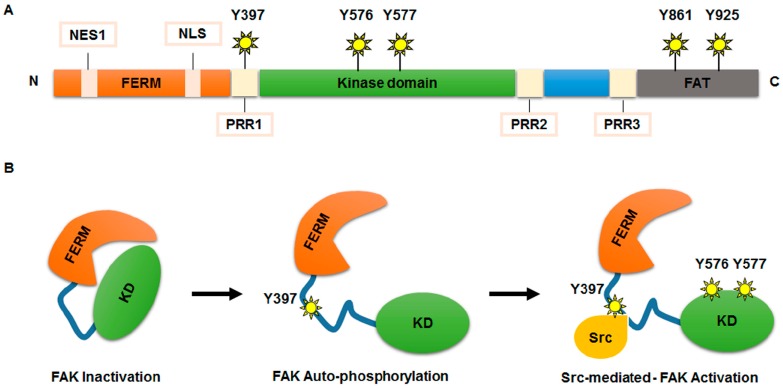
FAK protein structure and activation. (**A**) Schematic representation of the FAK protein structure. The N-terminal domain comprises a FERM domain, a nuclear export sequence 1 (NES1), a nuclear localization sequence (NLS) a proline-rich region (PRR1) and a 397-tyrosine auto-phosphorylation site (Y397). The central kinase domain contains Y576/Y577 phosphorylation sites, crucial for the kinase activity of FAK. The C-terminal domain includes a focal adhesion targeting (FAT) sequence and two proline regions (PRR2 and PRR3), which are important for binding with several molecular regulators and effectors. In C-terminal domain Y861 and Y925 phosphorylation sites are also included; (**B**) model of FAK activation. FERM domain binds to the central kinase domain maintaining FAK into an inactive form. Auto-phosphorylation at Y397 site removes FAK inhibition. Src kinase binds FAK at phosphorylation Y397 site generating a FAK-Src signalling complex, which contributes, after phosphorylation of Y576 and Y577 residues, to full activation of FAK activity.

The FAK protein comprises a multi-domain structure characterized by three main domains (N-terminal, central, and C-terminal) (Figure 1). The N-terminal domain contains the four-point-one, ezrin, radixin, moesin (FERM) domain, which consists of a nuclear export sequence 1 and a nuclear localization sequence (NLS) [23]. The FERM domain also includes binding sites for specific receptors or other interacting proteins (such as epidermal growth factor receptor, platelet derived growth factor receptor, c-Met, p53, and Ret) [23,24]. The central domain includes the kinase domain that is crucial for the activity of FAK [19]. In fact, the binding of the FERM domain to the central kinase domain locks FAK into its inactive state [19,23]. Finally, the C-terminal domain contains two proline-rich regions (PR2–PR3) and a focal adhesion targeting (FAT) region. These two sequences mediate the binding with several molecular regulators and effectors [22].

The functional activity of FAK is guaranteed by phosphorylation of several tyrosine (Y) residues: Y397 and Y407 at N-terminal domain, Y576 and Y577 within the central domain, and Y861 and Y925 at C-terminal domain [19]. Different stimuli, including receptor tyrosine kinases, intracellular pH changes, integrins recruitment to extracellular matrix (ECM), G protein-coupled receptors, and cytokine receptors, are able to remove the FAK auto-inhibition maintained by FERM domain, triggering FAK auto-phosphorylation at the Y397 site, necessary for its activation and recruitment at focal adhesions [17]. At this point, Src kinase, or acceptor proteins, can bind FAK at phosphorylated Y397, forming a FAK-Src signalling-complex, which contributes to full activation of FAK by phosphorylation of its Y576 and Y577 residues [25].

FAK plays a master role in organization and regulation of focal adhesion via its kinase-dependent and a kinase-independent functions [17]. Once activated, FAK promotes the recruitment of paxillin, a cytoskeletal and scaffold protein involved in the assembling of the focal adhesions to ECM [26]. In fact, the major role of FAK is the regulation of assembly and disassembly of focal adhesions, essential dynamic events for the control of cell motility and directional cell migration [27]. Moreover, FAK assures an efficient focal adhesion turnover through the binding with talin, thus controlling its proteolysis [28]. Some lines of evidence suggest that these catalytic activities of FAK may occur after a previous binding of the inactive FAK FERM domain to the actin nucleating protein Arp3 facilitating the recruitment of the Arp2/3 complex before the formation of mature focal adhesion [29]. In this model, FAK acts as a scaffold protein.

The function of FAK as a scaffold protein is mediated by FERM domain, which is involved in the transfer of information between the cell cortex and the nucleus [30]. Lim et al. [31] showed that FAK may translocate into the nucleus, where the protein may establish a direct interaction with p53 and Mdm2, thus enhancing Mdm2-dependent p53 ubiquitination/turnover, cell proliferation, and survival. For this function of nuclear FAK all of the three subdomains of FERM domain are required [32]. Indeed, the F2 subdomain includes the NLS that promotes nuclear translocation of the protein, the F1 subdomain is required for p53-FAK interaction and, finally, the F3 subdomain, which is involved in FAK-Mdm2 interaction [31,32,33]. On the other hand, Serrels et al. [34] demonstrated that nuclear catalytically active FAK interacts with chromatin and with transcription factors involved in intra-tumoural response of T regulatory cells in squamous cell carcinoma, thus suggesting that FAK may influence gene transcription and promote tumour immune escape.

### 2.2. Functions of FAK in Cancer

Noteworthy, FAK kinase activity is crucial for many cellular processes, which are associated with cancer cell growth and metastasis [35,36]. The FAK-dependent tumour cell processes include survival, proliferation, migration, invasion, epithelial-mesenchymal transition (EMT), and angiogenesis [35,36,37,38,39,40,41,42,43,44,45].

Numerous studies have documented an enhanced downstream signalling of FAK in cancer cell survival [17]. Indeed, the activation of FAK/Src complex converges in the up-regulation of signalling cascades, including PI3K-Akt, ERK1/2, and other mitogen-activated protein kinases, known to sustain cell survival by promoting cell resistance to “anoikis” (a death program for anchorage-dependent cells) under the condition of cell detachment, due to the disruption of adhesions between cells and ECM [37,38,39]. Moreover, a prominent role of FAK activity in the cell cycle progression was reported [40,41]. In particular, Zhao et al. [40] demonstrated that, in fibroblasts cell culture, FAK was able to regulate the expression of Cyclin D1 gene, a key regulator of G1/S phase progression of the cell cycle, via ERK1/2 pathway. A similar pro-proliferative role of FAK was described in glioblastoma cells [41]. In fact, in these cells the expression of a dominant negative mutant for FAK autophosphorylation caused the exit from G1 phase by reducing the expression of cyclins D1 and E, and enhancing the expression of p27Kip1 and p21Waf1 [41].

Interestingly, once in its active conformational state, FAK may mediate the dissolution of intercellular junctions, the up-regulation of mesenchymal markers, such as metalloproteinases (MMP)-2 and 9, and the down-regulation of membrane-bound E-cadherin, all molecular events associated with EMT and the predisposition to cell invasion and metastasis [42,43,44]. However, it is remarkable that FAK may affect E-cadherin expression in cancer cells by different mechanisms, suggesting that the pharmacological inhibition of FAK may counteract invasion and metastasis at least in cancers over-expressing PTK2 gene/protein [18].

Finally, a prominent role of FAK as key regulator of angiogenesis was also reported [45]. The authors found that blocking FAK activity in endothelial cells (ECs), by generating a conditional FAK-kinase-dead knock-in mouse model, prevented vascular endothelial growth factor (VEGF)-dependent vascular permeability. The study demonstrated that VEGF promotes tension-independent FAK activation, FAK localization to endothelial adherent junctions, binding of the FAK FERM domain to VE-cadherin, and direct FAK phosphorylation of β-catenin on Y142 residue, causing disassembly of junctions, supporting vascular permeability and tumour cell extravasation [45].

## 3. Role of FAK in HCC

### 3.1. FAK in Human HCC Samples

FAK over-expression and/or hyper-phosphorylation were described in many types of human cancer, including breast, colon, melanoma, thyroid, ovarian, and HCC [18]. However, the correlation of FAK over-expression with the disease stage, patient outcome, and prognosis was not investigated in most type of cancers except HCC.

So far, many studies have shown that FAK is over-expressed and hyper-phosphorylated in HCC tissues [46,47,48,49].

In 2001 Miyasaka et al. [46], using a combined approach of suppression subtractive hybridization, analysed the differences in gene expression between tumour and non-tumour liver tissues from 10 adult subjects with HCC developed in a viral background. This study identified seven differentially expressed genes, including FAK, which resulted up-regulated in HCC samples compared to normal livers [46]. Itoh et al. [47] extensively investigated the FAK protein over-expression in HCC in 64 patients who had undergone liver resection after tumour diagnosis without preoperative treatment. They found an association between FAK expression and clinic-pathological features of HCC. In particular, FAK was strongly up-regulated in HCCs with primary lesion and portal venous invasion, suggesting a role of this protein as prognostic factor in tumour progression [47]. In addition, an interesting study, investigated the expression of FAK mRNA and protein in 60 HCC human samples [48]. This study demonstrated that FAK was up-regulated at the level of mRNA and protein in tumour samples compared to matched non-tumour livers. Moreover, FAK gene over-expression was significantly correlated with high serum levels of alpha-fetoprotein and large tumour size, thus suggesting FAK mRNA levels as a potential predictor of tumour recurrence and overall survival [48]. All of these findings were further confirmed by Chen et al. [49], which found that total and phosphorylated forms of FAK were over-expressed in HCC tissues and correlated with tumour stage, vascular invasion, and intra-hepatic metastasis.

An increased expression of FAK in cancer could be dependent on: (i) mutation of the gene; (ii) epigenetic regulation of gene transcription; and (iii) post-translational modifications that regulate protein stability. In HCC, several studies demonstrated that FAK over-expression is mainly related to amplification of its gene (*PTK2*) [15,50,51,52,53]. Okamoto et al. [50], by using comparative genomic hybridization (CGH), provided the first evidence that regional amplification on chromosome 8q was common in HCC. The authors found in 39 primary HCCs that *PTK2* gene was amplified in 19% cases analysed accordingly to it is up-regulation [50]. The presence of *PTK2* gene amplification was confirmed by CGH performed on 19 surgically resected HCCs, revealing that this mutation was detected in 26% of cases and exclusively in moderately-differentiated and poorly-differentiated tumours [51]. A similar percentage (26.1%) of *PTK2* gene amplification was reported also by the whole-genome sequencing (WGS) demonstrating that this is one of the most prevalent and potentially actionable mutations in HCC [15]. However, a more recent WGS performed on 231 HCCs [52], as well as datasets available in cBioPortal for cancer genomics [53], showed that the amplification of the *PTK2* gene is lower with respect of that already reported, ranging from 1.3% to a maximum of 15.6%. Since FAK overexpression/activity was frequently reported in HCC from different aetiologies, apart from the gene mutation it is plausible that other still unknown mechanisms may explain FAK up-regulation in this type of cancer [46,47,48]. All of these findings highlighted that, independently of the frequency, gene amplification-dependent or -independent increases of *PTK2* gene expression could be crucial for a specific subset of HCCs that could benefit from therapeutic strategies against FAK expression/activity.

### 3.2. FAK in HCC Cells

As reported above, several lines of evidence suggested that FAK might be crucial for hepatocarcinogenesis. However, its real direct/indirect role in the control of HCC cell biology has only recently started to be explored. FAK appears to be essential for the regulation of the integrin-mediated adhesive and migratory properties of HCC cells. In fact, in 2010, Chen et al. [49] demonstrated that the siRNA-mediated knockdown of FAK in human HCC cell lines decreased cell adhesion and migration and concomitantly caused a significant reduction of both MMP-2 and MMP-9 expression and activities. Moreover, Gillory et al. [54] demonstrated that the inhibition of FAK phosphorylation/activation by silencing or by specific drug delivery caused a reduction of cell viability, invasion and migration of human hepatoblastoma HuH6 cells. The result was also confirmed in vivo in mice bearing subcutaneous HCC xenograft tumours. In addition, the hypothesis that FAK inhibition may impair HCC cell growth, mainly by inhibiting cell invasion and metastasis, was further confirmed by more recent in vitro and in vivo studies.

Von Sengbusch et al. [55] demonstrated that the down-regulation of FAK in HCC cells by using the dominant-negative of FAK-related non-kinase (FRNK) reduced metastatic adhesion within liver sinusoids in an in vivo model. Further experimental analyses demonstrated that the up-regulation of FAK expression in liver cancer is mediated by Argonaute2 (Ago2), a protein involved in miRNA maturation [56]. In this study, the authors showed that Ago2, found over-expressed in HCC cell lines, was able to induce cell proliferation and in vitro colony formation by transactivation of the FAK gene. Indeed, chromatin immunoprecipitation assay revealed that Ago2 binds regulatory regions of the FAK promoter [56].

In HCC, great emphasis has been given to the role of Notch1/Phosphatase and tensin homolog (PTEN) pathways in the activation FAK protein [57]. Different cellular functions have been assigned to Notch1 signalling, most of which refer to regulation of cell fate determination, including cell proliferation, differentiation, and programmed death [58]. On the other hand, PTEN, already known to be a potent tumour suppressor, negatively regulates FAK phosphorylation and activation [59,60]. Hu et al. [57] found that the Notch1 transcript was highly expressed in HCC cells compared to normal L02 liver cells. Interestingly, following Notch1 depletion, both HCC cell lines displayed impaired migration and invasion capability, increased protein expression of PTEN, and decreased expression of phosphorylated FAK. In the light of these findings, the authors hypothesized that the downregulation of Notch1 may inhibit HCC growth by up-regulating PTEN expression and consequently inactivating FAK [57].

Another molecular mechanism leading to FAK activation has been characterized during EMT occurring in hepatic stellate cells (HSCs), a crucial molecular event that precedes HCC development [61]. The authors found that the metalloproteinases inhibitor 1, (TIMP-1), secreted by tumour growth factor (TGF)-β1-activated HSCs, was able to mediate the crosstalk between HCC and HSC cells through FAK signalling. In particular, the interaction between TIMP-1 and CD63 on HCC cell surfaces was involved in the activation of the FAK pathway and in the increase of tumour cell proliferation, migration, and survival [61].

In 2015, an interesting study tried to elucidate the role of FAK in HCC by performing a hepatocyte-specific deletion of FAK in an oncogenic-induced mouse model of HCC obtained after c-MET and β-catenin (CAT) co-delivery [62]. Since no differences in viability and fertility were observed between liver FAK-depleted mice and wild type mice, they suggested that FAK is not required for normal liver development. On the contrary, the MET/CAT mice with the hepatic specific deletion of FAK developed small hepatic tumours compared to their wild-type counterpart. Furthermore, the BrdU staining of murine hepatocytes confirmed that FAK deficiency in a MET/CAT-driven HCC decreased cell proliferation and concomitantly reduced the activation of Akt and ERK1/2 proteins, main actors of molecular pathways driving cell proliferation [62]. Overall, this data suggests that anti-FAK therapy could be a suitable strategy in human HCC harbouring mutations in the β-catenin gene [62]. According to this data, a recent study demonstrated that the use of a specific inhibitor of tyrosine kinase activity of FAK, PF-562271, significantly suppressed MET/CAT-induced hepatocarcinogenesis [63]. Moreover, an in vitro and in vivo study, by Chung et al. [64], reported that Lipocalin enhanced migration and invasion abilities in liver cancer cells by promoting reduction of E-cadherin levels and increasing MET and FAK protein phosphorylation. Two additional studies, have also revealed that FAK phosphorylation/activation, and consequent HCC cell proliferation and invasion, are mediated by several pro-oncogenic signallings [65,66]. Among these pathways emerge the role of both Rab5a, a member of RAS oncogene family which is frequently overexpressed in HCC tissues in in vitro HCC experimental models [65], and of ERK5, which depletion blocks tumour growth in HCC xenografts and induces redistribution of FAK at focal contacts [66].

Finally, Zhang et al. [67], provided novel insights into the biological function of Galectin-1 signalling in HCC, demonstrating that the over-expression of this protein enhanced HCC invasion and sorafenib resistance by up-regulating the expression of αvβ3 integrin which, in turn, activated the FAK/PI3K/Akt pathway [67].

Several lines of evidence demonstrated that microRNAs (miR) have a fundamental role in HCC invasion and metastasis becoming possible diagnostic markers and therapeutic targets for tumour treatment [68,69]. Interestingly, FAK protein expression was significantly increased in HCC cells treated with miR-379-5p inhibitor. Moreover, the ectopic expression of miR-379-5p significantly reduced EMT, invasion, and metastasis in both in vitro and in vivo HCC by inhibiting FAK and the PI3K/Akt pathway [70].

## 4. FAK in Cancer Stem Cells: A Look at HCC

### 4.1. Cancer Stem Cells in HCC Pathogenesis

Traditionally, it has been assumed that the progressive accumulation of multiple genetic and epigenetic changes in mature hepatocytes lead to clonal evolution of tumour cells, resulting in the so-called stochastic (clonal) model for cancer development [71]. Currently, recent studies suggest that the heterogeneous nature of HCC morphology and behaviour may result from the hierarchical organization of different distinct lineages of hepatic cells, such as adult hepatocytes, hepatoblasts, and a subset of cells with stem and progenitor cell features, opening the way to the “cancer stem cell (CSCs) hypothesis” [72]. Due to their self-renewal and differentiated cell production capability, CSCs can support the tumour formation [71,72]. CSCs also exhibited elevated resistance to chemotherapeutic agents and are often responsible for tumour recurrence [73].

New insights into HCC development are gradually affirming the strong involvement of liver CSCs to maintain the hepatic tumorigenic properties [74]. Liver CSCs may originate from different lineages of hepatic cells: dedifferentiated cells that have acquired CSC characteristics, tumour hepatic progenitor cells and stem cells [75]. According to the CSC model, the extensive and chronic liver damage/inflammation may encourage the hepatocyte senescence and impair their self-renewal ability, stimulating the activation and proliferation of resident hepatic progenitor cells (HPCs) cells that may transform into CSCs [75].

Recent studies on CSC markers suggest that HCCs are heterogeneous and contain a subset of cells expressing a variety of common and hepato-specific markers, including epithelial cell adhesion molecule (EpCAM), OV6, CD133, CD90, CD44, CD24, CD13, and aldehyde dehydrogenase [75].

At the molecular level, aberrant activation of some signalling pathways (Wnt/β-catenin, Sonic Hedgehog, Notch, TGF-β1 and integrins), and altered expression of genes essential for maintenance of the stem cell phenotype (Oct4, Sox2, Nanog) have been frequently found to be associated to CSC activation [76,77]. These CSC-specific genes and other liver specific genes (Gankyrin) cooperate in hepatocarcinogenesis [78].

A recent paper by Sun et al. [75] demonstrated that Nanog over-expression in HCC cells positively correlated with tumour malignancy and metastatic cells features in both in vivo and in vitro models. The authors also found that over-expression of Nanog improved HCC cell invasion by activating EMT via SMAD2/3 protein phosphorylation [75].

Moreover, the co-expression of Nanog and Oct4 was found to be tightly associated with HCC progression and poor outcomes. In a HCC cell line with low metastatic potential cell clone (MHCC97L), Oct4 and Nanog sustained EMT and promoted migration and invasion through activation of the STAT3/Snail pathway [76].

Finally, more recently, You et al. [77] demonstrated that ECM stiffness was involved in the process of HCC stemness regulation via activating integrin β1/Akt/mTOR/Sox2 signalling. 

### 4.2. FAK and Stemness

Importantly, the close relationship between FAK and CSCs regulation has been well-documented in different tumour types [79,80,81]. Luo et al. [79] demonstrated that the mammary epithelial deletion of FAK in a MMTV-PyMT mouse model of breast cancer caused a decrease of CSCs pool. These CSCs exhibited reduced self-renewal and impaired migration capability in vitro. Moreover, the same CSCs displayed a less effective tumorigenicity when transplanted in NOD-SCID mice, suggesting that FAK was essential for the maintenance of CSC phenotype. Willliams et al. [80] found increased levels of phospho-FAK and major radiotherapy resistance in CSCs enriched from ductal carcinoma in situ of breast cancers compared to total cell population from the same tumours. In this pool of CSCs the inhibition of FAK phosphorylation resulted in a decrease of mammosphere formation and in a reduced ability to form tumours in xenografts through the downregulation of Wnt3a and Wnt activity [80]. An additional link between FAK and CSCs was reported in mesothelioma, where a potent inhibitor of FAK called VS-4718, reduced the CSC subset [81].

Additional reports highlighted a novel role of FAK in the control of the epidermal stem cell compartment. In the skin, the maintaining of stem cell properties is under the control of the Wnt/β-catenin pathway. Specifically, β-catenin regulates the stem cells localization at hair follicle inducing their mobilization and switch from a quiescent state to a proliferative state [82]. In a skin carcinogenesis mouse model, FAK may control β-catenin nuclear localization and transcriptional activity. At the same time, FAK expression is upregulated by β-catenin suggesting that there is a complex feedback loop between the two pathways [82].

**Figure 2:**
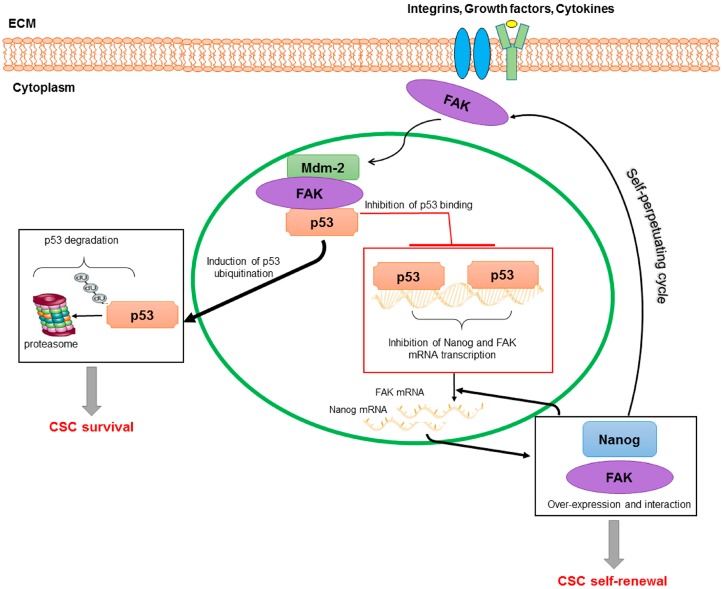
A model of regulation of CSCs based on p53/Nanog/FAK cross-linked-signalling. FAK may translocate into the nucleus where interacts with p53 and Mdm-2. This interaction causes p53 ubiquitination and consequent degradation via proteasome; and reduces p53 binding and repressive activity on Nanog and FAK promoters. In this way, Nanog and FAK over-expression, as well as their function and physical interaction, are self-perpetuated promoting CSC survival and self-renewal.

Finally, in colon and breast tumours, Ho et al. [83] showed that Nanog is recruited in the FAK promoter region at four binding sites, thus up-regulating PTK2 gene expression. At the same time, the authors reported that FAK and Nanog physically interact influencing Nanog phosphorylation/activity and its effects on cancer cell morphology, invasion, and growth [83]. Noteworthy, based on this study and previous studies demonstrating that the binding of p53 to Nanog and FAK promoter blocks the gene transcription [84,85], Golubovskaya, proposed an interesting molecular model of CSC regulation based on p53-Nanog-FAK cross-linked-signalling [86]. It is known that FAK can directly interact with p53, suppressing p53-trascriptional activity, but also with Mdm-2, causing ubiquitination of p53 and enhancing cell survival [86,87,88]. Thus, it is plausible that in a condition of FAK over-expression, like that observed in HCC, a down-regulation of p53 repression activity on both FAK and Nanog promoters may occur. This may promote a vicious circle in which FAK and Nanog over-expression is self-perpetuated (Figure 2). Therefore, it is expected that targeting the FAK/Nanog/p53 network with small molecules could contribute to up-regulate p53 activity with consequent apoptosis of both adult cancer cells and CSCs. Moreover, simultaneous addition of the drugs targeting these interactions can decrease tumour growth through inhibition of tumour cell survival.

### 4.3. FAK in HCC CSCs

Scientific evidence on the role of FAK on HCC CSCSs is still sparse and based on little experimental data. Park et al. [61] reported that FAK signalling might regulate the CSC features of HCC cells. In particular, the authors demonstrated that FAK inhibitor-14 (FAKI-14) significantly reduced sphere formation and CD44/CD90 expression, and decreased the mRNA expression of OCT4, NANOG, SOX2, KLF4, and C-MYC in the SK-HEP1 HCC cell line.

Furthermore, a recent study demonstrating that ankyrin-repeat-containing, SH3-domain-containing, and proline-rich-region-containing protein 2 was able to suppress stem cell-like characteristics and chemoresistance in HCC by the inhibition of the Src/FAK/Snail axis [89].

These pieces of data highlights that understanding FAK signalling in HCC stemness deserve further investigation that could provide future anti-cancer therapy approaches.

## 5. FAK Inhibitors in Clinical Applications

### 5.1. FAK Inhibitors

In recent years, a discrete number of studies on FAK inhibition have been carried out in order to investigate FAK as a promising therapeutic target for cancer [90,91]. Studies of in vitro and in vivo cancer models have shown an enhanced anti-tumoural effect of FAK inhibition when delivered in combination with cytotoxic drugs or agents that targeted angiogenesis, such as the receptor tyrosine kinase inhibitors. Pharmaceutical companies have designed several drugs that may inhibit FAK. These drugs are prevalently small molecules that can be grouped into kinase inhibitors that block FAK catalytic activity by ATP-binding or by alternative means, and compounds targeting FAK scaffold functions [17,90]. TAE226, PF-562271, PF-573228, and PND-1186, which belong to the group of ATP-competitive kinase inhibitors, interact with residues surrounding the ATP binding pocket of the protein inhibiting its activity [92,93,94]. Since inhibitors that function as ATP analogues may also target the ATP binding pocket of other tyrosine kinases reducing their specificity, new drugs directly interfering with FAK autophosphorylation have been developed, such as Y15 and Y11 [95,96].

Some of the mentioned FAK inhibitors are currently under evaluation in Phase I and II clinical trials in patients with solid tumours, but for the final results we will have to wait [17,90].

### 5.2. FAK Inhibitors in HCC Models

There are several treatment strategies available for HCC, including surgical resection, transarterial chemoembolization, radiofrequency ablation, and percutaneous ethanol injection and liver transplantation [97]. More recently, the identification of mechanisms that play a crucial role in HCC pathogenesis, such as neoangiogenesis, has led to development of systemic targeted therapies [98]. However, currently sorafenib, a multi-targeted tyrosine kinase inhibitor, is the only systemic agent found to increase survival time in patients with locally advanced and/or metastatic HCC who are not candidates for either resection or liver transplantation and have failed to respond to locoregional therapies [99]. Unfortunately, patients with HCC receiving sorafenib may develop drug resistance due to crosstalk with different signalling molecules, including FAK [100,101]. Thus, other targeted therapies are currently under evaluation [99].

Some of the above mentioned pharmaceutical inhibitors of FAK were also recently used in HCC in vivo models. In a rat HCC xenograft model, which was obtained by subcutaneous injection of Huh7.5 HCC cells, Bagi et al. [102] evaluated the anti-tumour effect of sunitinib and PF-562271 combination therapy. The results of the study well-demonstrated that appropriate combination of sunitinib and PF-562271 was able to block not only tumour growth, but also to impair HCC recovery upon withdrawal of therapy [102]. Moreover, it was reported that PF-562271 alone, decreased the FAK phosphorylation and concomitantly reduced the invasive and migratory ability of HCC cells through inhibiting MMP-2 and MMP-9 expression and activity [103]. As already mentioned the PF-562271 may also reduce size of tumour in a MET/CAT-driven HCC [63].

Gillory et al. [54] showed that PF-573228 reduced FAK phosphorylation in human hepatoblastoma cells, thus impairing in vitro cell viability, invasion, migration, and attachment-independent growth, and increasing apoptosis. Similar effects were observed in in vitro and in vivo models of hepatoblastoma by using Y15, which caused a significant reduction of the phosphorylated-FAK/FAK ratio [54].

In a very recent study, Wang et al. [104] reported synergic anti-tumour effects of cabozantinib, a MET inhibitor, in combination with a novel FAK inhibitor (CT-707). This combination therapy synergistically reduced in vitro and in vivo HCC growth by counteracting the cabozantinib-dependent activation of FAK [104].

In addition to pharmacological inhibitors, also natural molecules able to inhibit FAK have been recently used in HCC models [103,105,106]. These natural compounds, including 3′3-diindolylmethane, sinulariolide, and corosolic acid, are not specific for FAK, but they can inhibit HCC multiple signalling pathways [103,105,106].

## 6. Conclusions

The clinical management of HCC is complicated by the heterogeneity of this tumour type, which may render current therapeutic options ineffective. The different drugs against putative targets, including agents blocking tyrosine kinase receptors and their signalling pathways, cell growth and cell migration controllers, angiogenesis, or turnover of the proteins, may offer promising alternative therapeutic strategies. However, among these agents, there is still a lack of drugs or approaches for individual targeting of the different HCC phenotypes. The most common molecular signature in HCC is represented by the progressive accumulation of genetic and epigenetic changes in mature hepatocytes. However, recent studies suggest that heterogeneous nature of this type of tumour originates from different distinct intra-hepatic cell lineages, such as mature hepatocytes, and subset of cells with stem and progenitor cell features that may be pivotal in HCC recurrence.

Novel targetable gene/molecular alterations, known as clinically actionable, such as the regulation of FAK overexpression and activity, could reveal novel drug candidates against HCC. Small molecules with FAK inhibitory properties are emerging as promising chemotherapeutics, inhibiting growth, metastasis, and angiogenesis in different types of tumours [17]. Unfortunately, similarly to the other tyrosine kinase inhibitors, FAK inhibitors may suffer from several drawbacks [107]. In fact, resistance that can be developed in several ways often accompanies their use. Moreover, if they are not specific they may also act on other kinases, consequently leading to unpredictable side effects, and could affect normal cell homeostasis. Finally, as most of the studies have been performed in immunocompromised mice we do not know their immunomodulatory effects. However, very recently, it has been reported that FAK silencing successfully decrease HCC cell resistance to sorafenib [101]. This last evidence, and the recent studies on the role of FAK in HCC reviewed here, supports the hypothesis that FAK inhibitors could be effective as adjuvant therapies in HCC management.
